# E-cadherin signal sequence disruption: a novel mechanism underlying hereditary cancer

**DOI:** 10.1186/s12943-018-0859-0

**Published:** 2018-08-01

**Authors:** Joana Figueiredo, Soraia Melo, Kimberley Gamet, Tanis Godwin, Susana Seixas, João M. Sanches, Parry Guilford, Raquel Seruca

**Affiliations:** 1 Instituto de Investigação e Inovação em Saúde (i3S), Porto, Portugal; 2 Institute of Molecular Pathology and Immunology of the University of Porto (IPATIMUP), Porto, Portugal; 3 Medical Faculty of the University of Porto, Porto, Portugal; 4 Genetic Health Service NZ, Auckland City Hospital, Auckland, New Zealand; 5 Cancer Genetics Laboratory, Centre for Translational Cancer Research (Te Aho Matatū), Department of Biochemistry, University of Otago, Dunedin, New Zealand; 6 Institute for Systems and Robotics (ISR/IST), LARSyS, Bioengineering Department, Instituto Superior Técnico, Universidade de Lisboa, Lisbon, Portugal

**Keywords:** Hereditary Diffuse Gastric Cancer, E-cadherin, *CDH1*, Germline variants, Signal peptide, Post-translational mechanism

## Abstract

The aim of this study was to uncover the pathogenic relevance and the underlying molecular mechanism of a novel *CDH1* variant found in a Hereditary Diffuse Gastric Cancer family (p.L13_L15del), which affects the signal peptide of E-cadherin without changing the remaining predicted sequence. We verified that p.L13_L15del cells yield low levels of E-cadherin, decreased cell adhesion and enhanced cell invasion. Further, we demonstrated that the disruption of the highly conserved hydrophobic core of the signal peptide hampers the binding of cellular components crucial for E-cadherin translation and translocation into the endoplasmic reticulum, constituting a new molecular basis for the loss of a tumour suppressor gene causative of hereditary cancer.

**Electronic supplementary material:**

The online version of this article (10.1186/s12943-018-0859-0) contains supplementary material, which is available to authorized users.

## Main text

To date, more than 155 loss-of-function *CDH1* mutations have been described in Hereditary Diffuse Gastric Cancer (HDGC) [1]. The most common alterations induce the occurrence of premature termination codons with an obvious deleterious effect [2].

The mature E-cadherin, encoded by the *CDH1* gene, is a powerful adhesion molecule that contains a long extracellular domain responsible for the homophilic binding to cadherins presented on neighbouring cells, a transmembrane domain, and a cytoplasmic portion that supports the assembly of catenins and their anchorage to the cytoskeleton [3]. Importantly, before protein processing, the immature molecule also encompasses a short signal peptide and a precursor region preceding the extracellular domain [3]. Signal peptides serve as docking sites for the signal recognition particle (SRP), the main molecule responsible for detecting the translocation code of secretory and membrane proteins [4, 5].

Despite the remarkable biological function of the signal peptide, genetic changes occurring in this region are often ignored. The present study reports a novel *CDH1* germline variant found in a HDGC family, which affects the signal peptide core of E-cadherin and maintains an intact mature protein.

## Results and discussion

### Description of the family

**Fig. 1:**
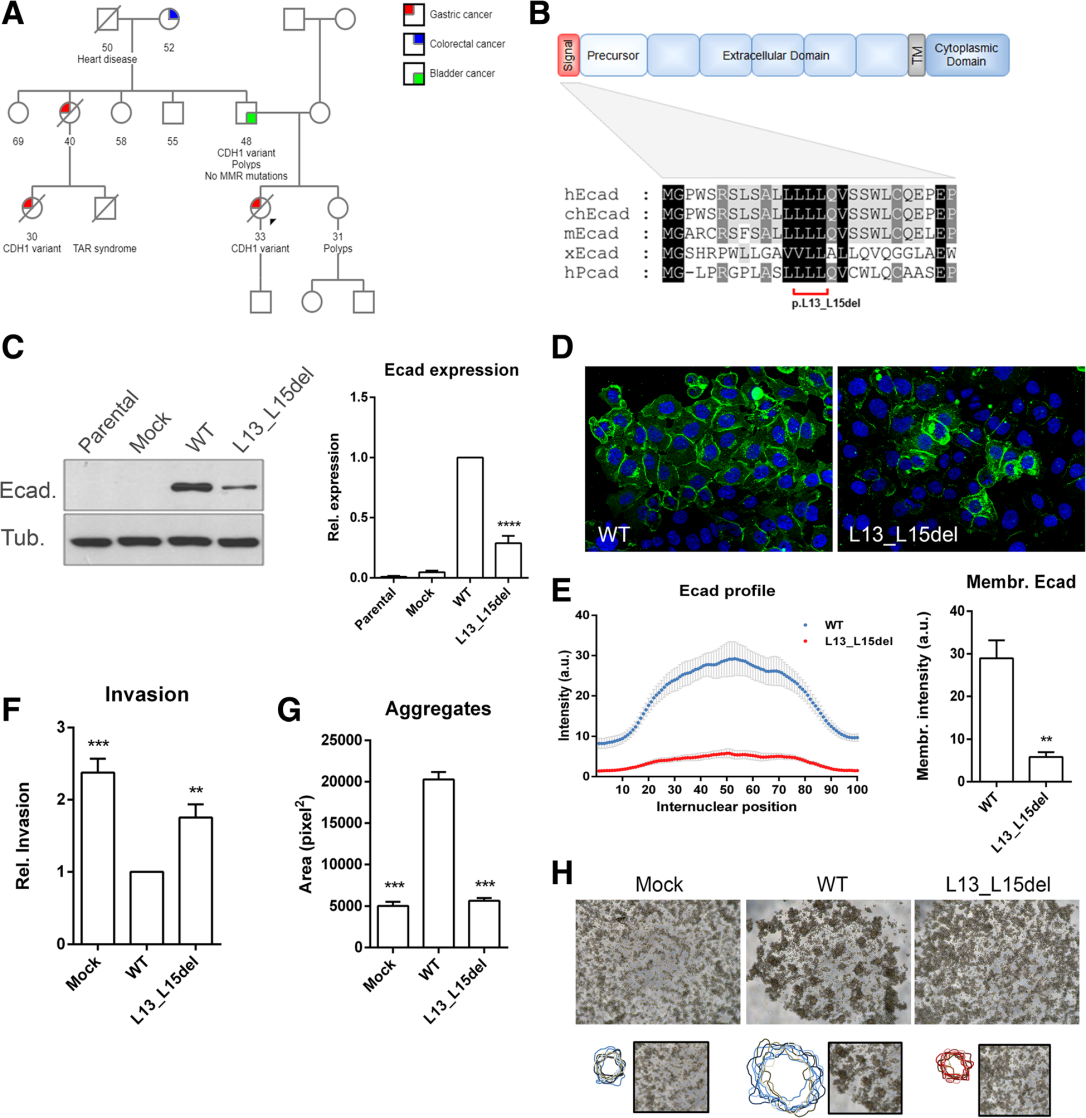
Description and functional characterization of p.L13_L15del variant. **a** The pedigree of a New Zealand family carrying the p.L13_L15del mutation is represented. Symbols with a slash indicate deceased individuals. The proband is identified with the arrowhead. Cancer or other known diseases affecting family members were indicated. The actual age or the age at the time of death is displayed below each individual. TAR denotes thrombocytopenia-absent radius syndrome and MMR stands for mismatch repair. **b** Schematic representation of E-cadherin comprising the signal peptide, precursor, extracellular, transmembrane and cytoplasmic domains. Multiple sequence alignment of five signal peptide sequences is shown (hEcad, human E-cadherin; chEcad, chimpanzee E-cadherin; mEcad, mouse E-cadherin; xEcad, *Xenopus* E-cadherin; hPcad, human P-cadherin). Conserved residues are highlighted in black boxes, and residues conserved in at least four cadherins are shown in dark grey. **c** Total levels of E-cadherin were analyzed by Western Blot in CHO cells transfected with vectors encoding the E-cadherin mutant p.L13_L15del, the wild-type protein, and the empty vector (Mock). α-Tubulin was used as a loading control. Band intensity was quantified and normalized against wild-type cells. Intensity average + SE is represented in the graph. **d** Immunofluorescence was applied to evaluate protein localization. E-cadherin is shown in green and nuclei were counterstained with DAPI (blue). **e** Expression profiles of mutant (red) and wild-type cells (blue) were quantified. Average intensity in each internuclear position + SE is represented in the graph. Mean and SE of fluorescence intensity at the plasma membrane (internuclear position 50) is presented. **f** Invasive ability mean of wild-type and p.L13_L15del mutant cells. **g** Average area + SE of aggregates. **h** Cell-cell aggregation phenotypes of the different cell lines. Representative outlines of wild-type and mutant cellular aggregates are presented on the bottom. ** represents *p* ≤ 0.01, *** *p* ≤ 0.001 and **** *p* ≤ 0.0001

The heterozygous germline mutation c.38_46del, leading to the amino acid deletion p.L13_L15del, was identified by direct sequencing in a 33-year old woman from New Zealand (patient A). Histological examination of gastric specimens revealed that the proband was affected by signet ring cell (diffuse) carcinoma. One paternal aunt and a cousin were diagnosed with the same type of neoplasia and died at 40 and 30 years of age (Fig. 1a). The affected cousin, as well as patient A’s father were carriers of the same genetic alteration. Of note, this mutation was not found in the largest database of human genetic variation to date (gnomAD: The Genome Aggregation Database) comprising several thousand of unrelated individuals [6].

### P.L13_L15del variant induces decreased total and membrane E-cadherin expression

To determine the pathogenicity of the L13_L15del *CDH1* mutation, we first studied the conservation of the signal peptide. We performed a multiple sequence alignment of the E-cadherin amino acid sequence from different species and of P-cadherin, given the similarity of both cadherins with respect to their cell-to-cell adhesive function and epithelial expression [3]. Although most of the sequence is variable, the signal hydrophobic core is highly conserved across different cadherins (Fig. 1b). The p.L13_L15del mutation affects this specific region by removing three amino acids from the six that comprise the hydrophobic region, which suggests its possible functional relevance. Accordingly, PROVEAN software predicts a deleterious effect for this mutation with a score of − 6.102 (score ≤ − 2.5 is considered deleterious, Additional file 1: Table S1). A putative effect on the signal peptide cleavage was also tested and, while the predicted cleavage site of the wild-type and the variant sequences remains unchanged, the probability of the variant to generate a functional signal peptide is greatly decreased (Additional file 2: Figure S1). In addition, we could confirm p.L13_L15del mutation as “likely affecting signal peptide quality” through the inhibition of protein translocation to the endoplasmic reticulum (ER) membrane (*min*(*∆C*)= − 0.321 > *min*(*∆S*)= − 0.430) [7].

To assess the impact of the p.L13_L15del alteration in vitro, we transfected E-cadherin negative cells with vectors encoding the wild-type protein and the variant, as well as the empty vector, as a control (Additional file 3: Methods and Materials). Despite similar transfection efficiencies in all conditions, we verified that total protein levels were significantly reduced in the mutant cells, when compared with the wild-type expressing cells (Fig. 1c). An abnormal pattern of E-cadherin localization was also detected by immunofluorescence in most of the cells. In contrast to the strong membrane staining presented by the great majority of the wild-type cells, the p.L13_L15del cells displayed very low levels of E-cadherin at the membrane and, occasionally, aberrant cytoplasmic accumulation of the protein (Fig. 1d). Quantitative evaluation of the staining showed more intense E-cadherin in the wild-type cells when compared with those expressing the p.L13_L15del (Fig. 1e). This difference is also reflected in fluorescence intensity exhibited at the plasma membrane.

Taken together, these results indicate that the p.L13_L15del variant affects a conserved region of the E-cadherin signal peptide and impacts protein expression (the total level and the membrane fraction).

### p.L13_L15del variant affects the adhesive and anti-invasive function of E-cadherin

To investigate the functional significance of p.L13_L15del variant, we tested cell invasive properties and cell-to-cell adhesiveness (Fig. 1f-h). In contrast to the transfection of wild-type E-cadherin, which significantly decreases the number of invasive cells, the variant is not able to efficiently suppress invasion through a matrigel matrix. Regarding adhesion, wild-type cells form large and compact aggregates with an average area of 20,277 pixels^2^, contrasting with p.L13_L15del expressing cells, which exhibit an isolated appearance with cellular structures of 5662 pixels^2^. Overall, our findings strongly support the pathogenic nature of the p.L13_L15del variant.

### P.L13_L15del does not induce abnormal protein trafficking or premature degradation

**Fig. 2:**
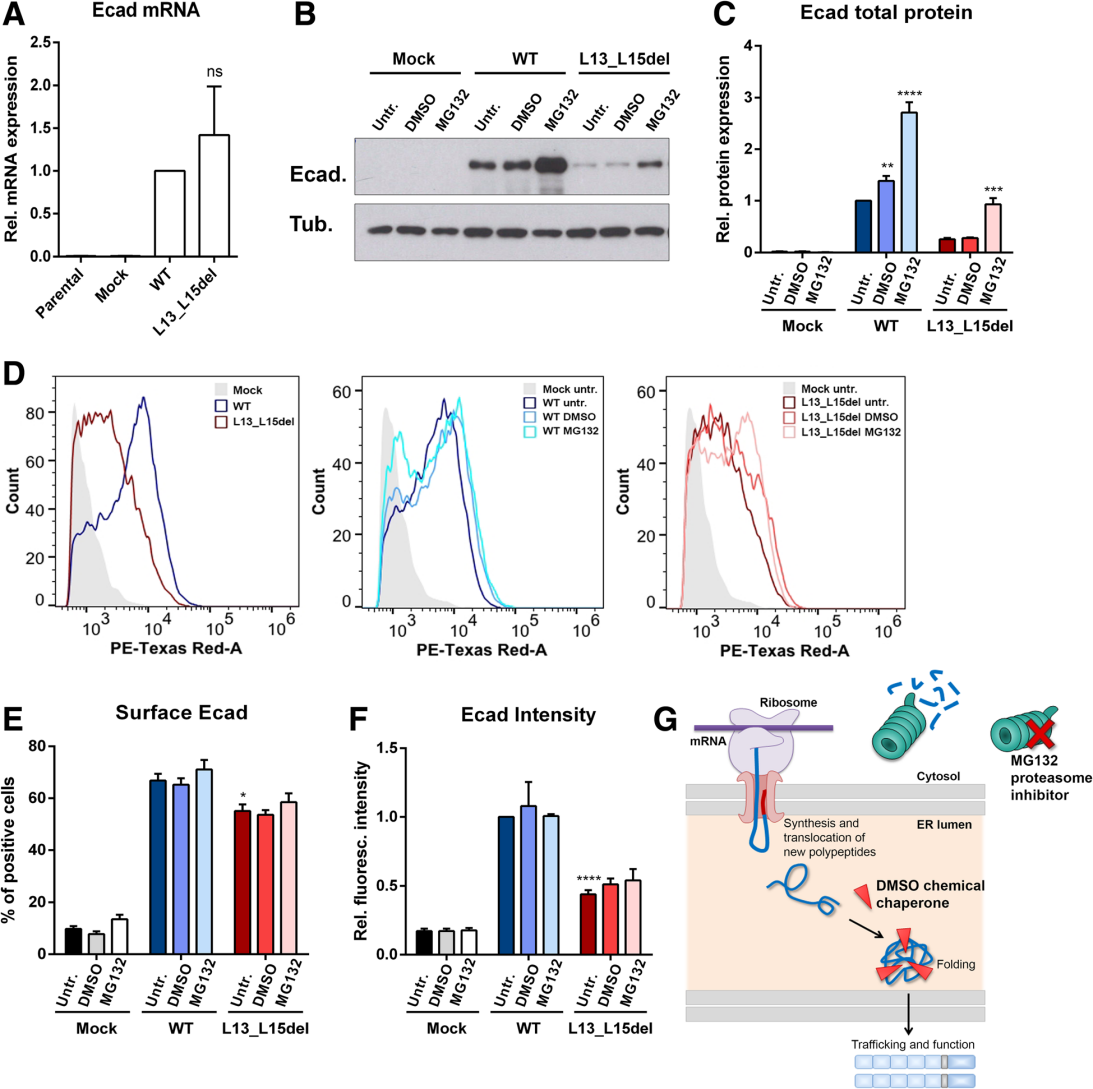
p.L13_L15del variant does not affect E-cadherin trafficking or degradation. **a** *CDH1* and *18S* mRNA levels were analyzed by real-time PCR in CHO cells transfected with vectors encoding the E-cadherin mutant L13_L15del, the wild-type protein, and the empty vector (Mock condition). 18S was used as endogenous control. **b** Total levels of E-cadherin were analyzed in Mock, wild-type and L13_L15del mutant cells upon treatment with 2% DMSO and 10 μM MG132. α-Tubulin was used as a loading control. **c** Band intensity average + SE is presented. **d** Flow cytometry was used to assess surface E-cadherin in cells untreated and treated with DMSO or with the proteasome inhibitor MG132. **e** Graph representing the percentage of cells positive for E-cadherin. **f** For each sample, median fluorescence intensity + SE was determined and normalized for untreated wild-type cells. * represents *p* ≤ 0.05, ** *p* ≤ 0.01, *** *p* ≤ 0.001 and **** *p* ≤ 0.0001. **g** Scheme illustrating the interaction of a chemical chaperone with a newly synthesized polypeptide at the lumen of the endoplasmic reticulum. The chemical chaperone assists the folding of the protein and evades its quality control and degradation. At the cytoplasm, MG132 blocks the proteolytic activity of the 26S proteasome complex, resulting in accumulation of immature and unfolded proteins

Finally, we determined the molecular mechanism underlying the deleterious effects of p.L13_L15del at the signal peptide of E-cadherin. Possible alterations at the RNA level were first evaluated by real-time PCR. We verified that *CDH1* mRNA levels were not significantly changed both in wild-type and in the variant conditions, despite the huge difference at the protein level (Fig. 2a). To exclude protein trafficking deregulation or premature degradation by quality control mechanisms, we treated the cells with DMSO chemical chaperone and MG132 proteasome inhibitor. Upon DMSO treatment, no effect was detected in either E-cadherin total expression, number of E-cadherin positive cells, or staining intensity of mutant cells (Fig. 2b-f). Proteasome inhibition induced a significant increase in p.L13_L15del protein levels and a slight increase in the number of cells expressing E-cadherin, as well as in the number of molecules present at the membrane. However, these increased levels were significantly different from those observed in the wild-type cells.

With this set of experiments, we demonstrated that the deleterious effect of p.L13_L15del is not dependent on protein trafficking deregulation or on its early degradation.

### P.L13_L15del hampers E-cadherin translation and processing

**Fig. 3:**
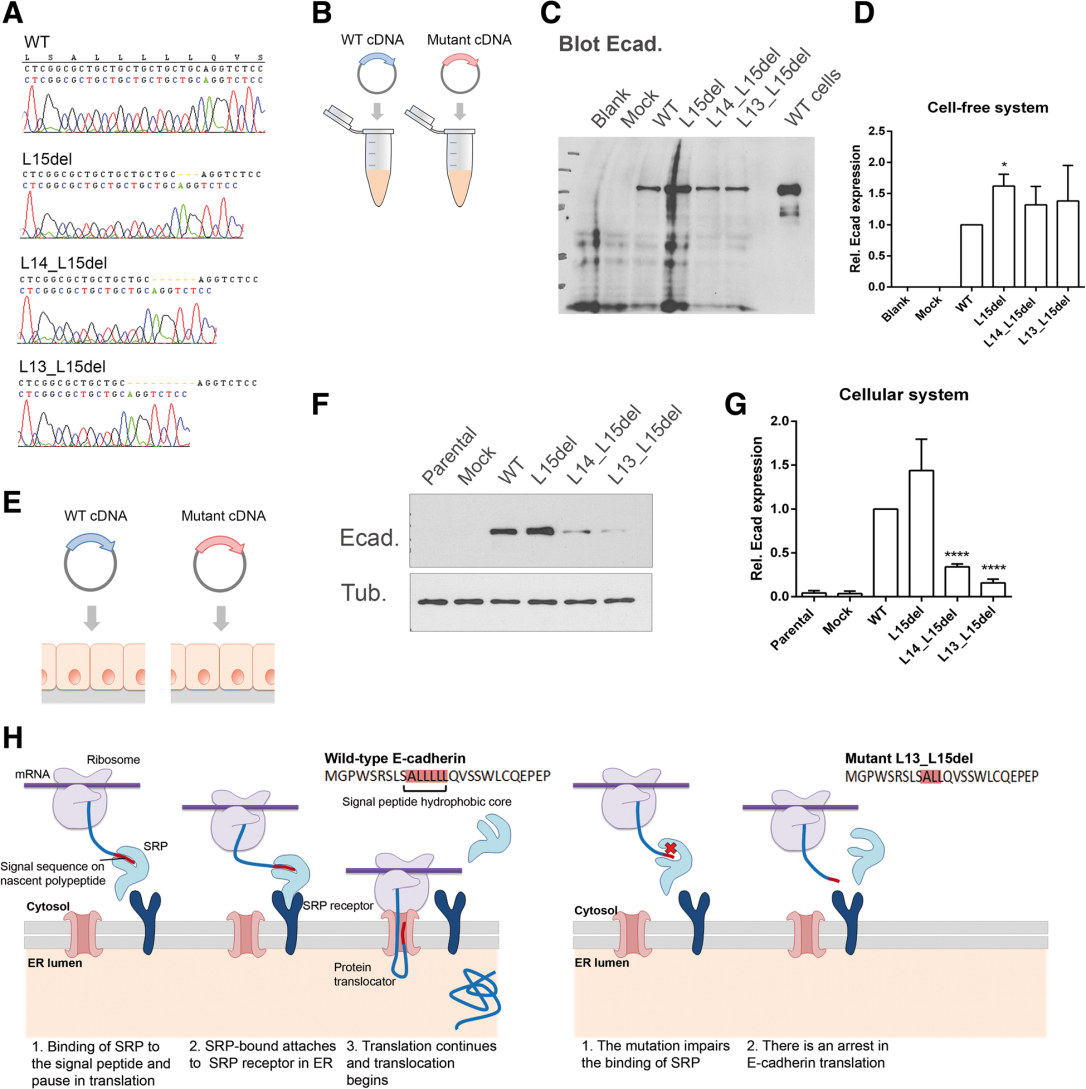
The p.L13_L15del E-cadherin mutant is regulated by cellular post-translational mechanisms. **a** cDNA sequences of mutant plasmids inducing the deletion of one, two or three leucine residues. **b** Representative scheme of the cell-free system for in vitro protein translation. Constructs encoding the wild-type and the mutant *CDH1* cDNAs were used as templates for the production of E-cadherin molecules. **c** Coupled transcription and translation of E-cadherin was detected by Western blotting. **d** Band intensity was quantified and normalized against wild-type plasmid. Intensity average + SE is represented in the graph. **e** Illustration of the cellular model: E-cadherin negative cells were transfected with plasmids encoding the wild-type and the sequential *CDH1* mutants. **f** E-cadherin levels produced by cells transfected with the p.L15del, the p.L14_L15del and the p.L13_L15del mutants. α-Tubulin was used as a loading control. **g** Quantification of band intensity is showed in the graph. (**h**) Regulation mechanism of E-cadherin mutant p.L13_L15del. In a wild-type context, the binding of signal recognition particle (SRP) to the signal peptide sequence of the nascent polypeptide causes a temporary pause in translation. Subsequently, SRP-bound ribosome attaches to SRP receptor located at the membrane of the endoplasmic reticulum, enabling translation to continue and translocation to begin. SRP is released and SRP receptor is recycled. The newly synthesized protein undergoes folding and trafficking to its correct location at the plasma membrane. The presence of the p.L13_L15del mutation can, however, impair the binding of SRP and, consequently, induce an evident arrest of E-cadherin translation, which is associated with several deleterious cellular effects

To test the involvement of post-translational machinery in the regulation of the p.L13_L15del mutant, we set up a cell-free system for in vitro protein translation (Fig. 3b). The approach allows coupled transcription and translation of a specific DNA sequence without the action of intermediate and regulatory cellular moieties. Vectors encoding the wild-type and the mutant *CDH1* cDNAs were used as templates for the production of E-cadherin molecules. E-cadherin synthesis was subsequently detected by immunoblot and it was verified that the wild-type and the p.L13_L15del plasmids produce similar amounts of the protein, in contrast to the clear difference observed in the cell model system (Fig. 3c-g).

To determine the impact of the specific leucine residues from the hydrophobic core in protein translation, a series of mutants was engineered. The sequential deletion of one, two or three leucine residues produces a decreasing effect on the quantity of E-cadherin molecules that are translated by the cells and that are exposed at the cell surface (Fig. 3f-g and Additional file 4: Figure S2). Concordant results were obtained by in silico analysis of these mutants, where the p.L13_L15del always displayed stronger deleterious effect than the p.L14_L15del, and the p.L15del behaved as a nearly neutral variant (Additional file 1: Table S1). Remarkably, in the cell-free system, no decreasing effects were observed in protein levels of any of the mutants when compared with the wild-type condition. These findings demonstrated that p.L13_L15del impairs the interaction of E-cadherin with post-translational machinery, decreasing protein synthesis, ER import and membrane activity. Further, we verified that the impairment of protein translation is dependent on the extension of the signal peptide core disruption.

Interestingly, two different germline mutations affecting the signal peptide core and which do not induce truncated *CDH1* forms were previously identified in diffuse gastric cancer cases: c.44_46del (p.L15del) and c.46insTGC (p.L15dup) [8–10]. Taking into account our results, both mutations are unlikely to cause HDGC. In fact, we demonstrated that p.L15del does not affect the function of the signal peptide, and in the case of p.L15dup, the integrity of the signal peptide core is preserved, which is indicative of normal translation and translocation into the ER.

In summary, this is the first description that the *CDH1* signal peptide core is essential for E-cadherin synthesis and delivery. The failure in this checkpoint leads to loss of protein expression and function, and ultimately to disease.

## Additional files

Additional file 1: **Table S1.** In silico prediction of the putative impact of E-cadherin variants. The results from PROVEAN and *R*-score predictions for the different E-cadherin forms are presented. For PROVEAN, variants generating a score equal or below − 2.5 were classified as deleterious, whereas for *R*-score a value above the 0.30 threshold was considered as deleterious. In the *R*-score, a *min*(*∆S*) << 0 indicates a decrease in signal peptide quality, a *min*(*∆C*) << 0 points to a loss of signal peptide cleavage site and a *min*(*∆C*) > *min*(*∆S*) can be interpreted as an evidence for translocation inhibition [7]. (DOCX 16 kb)

Additional file 2: **Figure S1.** Prediction of the signal peptide cleavage. Graphical output and summary of SignalP 4.1 predictions for the wild-type (A) and the p.L13_L15del (B) sequences. C-score distinguishes signal peptide cleavage sites. S-score discriminates amino acids constituting signal peptides from amino acids composing the mature form of the protein. Y-score combines C and S scores, generating an improved cleavage site prediction. D-score is the weighted average of the mean S and the maximum Y scores, differentiating signal from non-signal peptides. (TIF 869 kb)

Additional file 3: Methods and Materials. (DOCX 44 kb)

Additional file 4: **Figure S2.** E-cadherin surface expression induced by the p.L15del, p.L14_L15del and p.L13_L15del variants. (A) Histogram showing E-cadherin surface expression in cells transfected with plasmids encoding the wild-type or the L15del, L14_L15del and L13_L15del E-cadherin mutant forms. Mock cells were used as a negative control. (B) Percentage of cells expressing E-cadherin at the plasma membrane. The graph shows the average + SE of four independent experiments. (C) The relative median fluorescence intensity was determined in each cell line. * represents *p* ≤ 0.05, ** *p* ≤ 0.01, *** *p* ≤ 0.001 and **** *p* ≤ 0.0001. (TIF 496 kb)

## Abbreviations

DMSO: Dimethyl sulfoxide
ER: Endoplasmic reticulum
HDGC: Hereditary Diffuse Gastric Cancer
SRP: Signal recognition particle
