# Supplementary material for: E-cadherin signal sequence disruption: a novel mechanism underlying hereditary cancer
Source: Mol Cancer. 2018 Aug 1;17:112. doi: 10.1186/s12943-018-0859-0 (PMC6090902; doi:10.1186/s12943-018-0859-0)
Supplement: Supplementary file 1 — Table S1. In silico prediction of the putative impact of E-cadherin variants. The results from PROVEAN and R-score predictions for the different E-cadherin forms are presented. For PROVEAN, variants generating a score equal or below − 2.5 were classified as deleterious, whereas for R-score a value above the 0.30 threshold was considered as deleterious. In the R-score, a min(∆S) << 0 indicates a decrease in signal peptide quality, a min(∆C) << 0 points to a loss of signal peptide cleavage site and a min(∆C) > min(∆S) can be interpreted as an evidence for translocation inhibition [7]. (DOCX 16 kb) [file 12943_2018_859_MOESM1_ESM.docx]

**Table S1. *In silico* prediction of the possible impact of E-cadherin variants.** The results from PROVEAN and *R*-score predictions for the different E-cadherin forms are presented. For PROVEAN, variants generating a score equal or below -2.5 were classified as deleterious, whereas for *R*-score a value above the 0.30 threshold was considered as deleterious. In the *R*-score, a *min*($\Delta S)$ << 0 indicates a decrease in signal peptide quality, a *min*($\Delta C)$ << 0 points to a loss of signal peptide cleavage site and a *min*($\Delta C)$ > *min*($\Delta S)$ can be interpreted as a evidence for translocation inhibition [^20^](#_ENREF_20).

| Variant | PROVEAN | *R*-score | *min*$\boldsymbol{(\Delta S)}$ | *min*$\boldsymbol{(\Delta C)}$ |
| --- | --- | --- | --- | --- |
| L13_L15del | -6.102 | 0.588 | -0.430 | -0.321 |
| L14_L15del | -4.818 | 0.411 | -0.205 | -0.248 |
| L15del | -2.995 | 0.269 | -0.061 | -0.212 |
