# Supplementary figures and images for: E-cadherin signal sequence disruption: a novel mechanism underlying hereditary cancer
Source: Mol Cancer. 2018 Aug 1;17:112. doi: 10.1186/s12943-018-0859-0 (PMC6090902; doi:10.1186/s12943-018-0859-0)

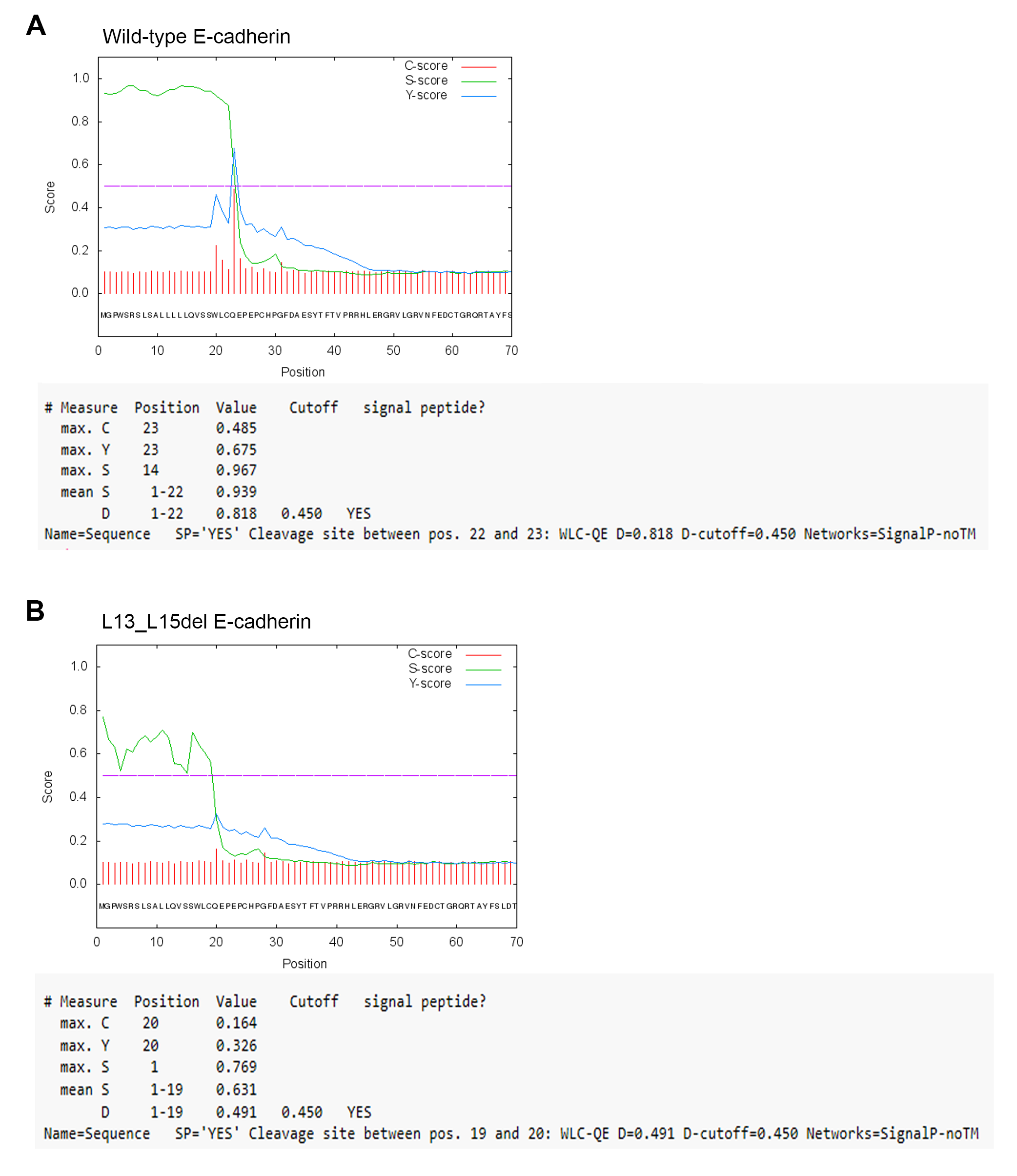

Supplement: Supplementary file 2 — Figure S1. Prediction of the signal peptide cleavage. Graphical output and summary of SignalP 4.1 predictions for the wild-type (A) and the p.L13_L15del (B) sequences. C-score distinguishes signal peptide cleavage sites. S-score discriminates amino acids constituting signal peptides from amino acids composing the mature form of the protein. Y-score combines C and S scores, generating an improved cleavage site prediction. D-score is the weighted average of the mean S and the maximum Y scores, differentiating signal from non-signal peptides. (TIF 869 kb) [file 12943_2018_859_MOESM2_ESM.tif]

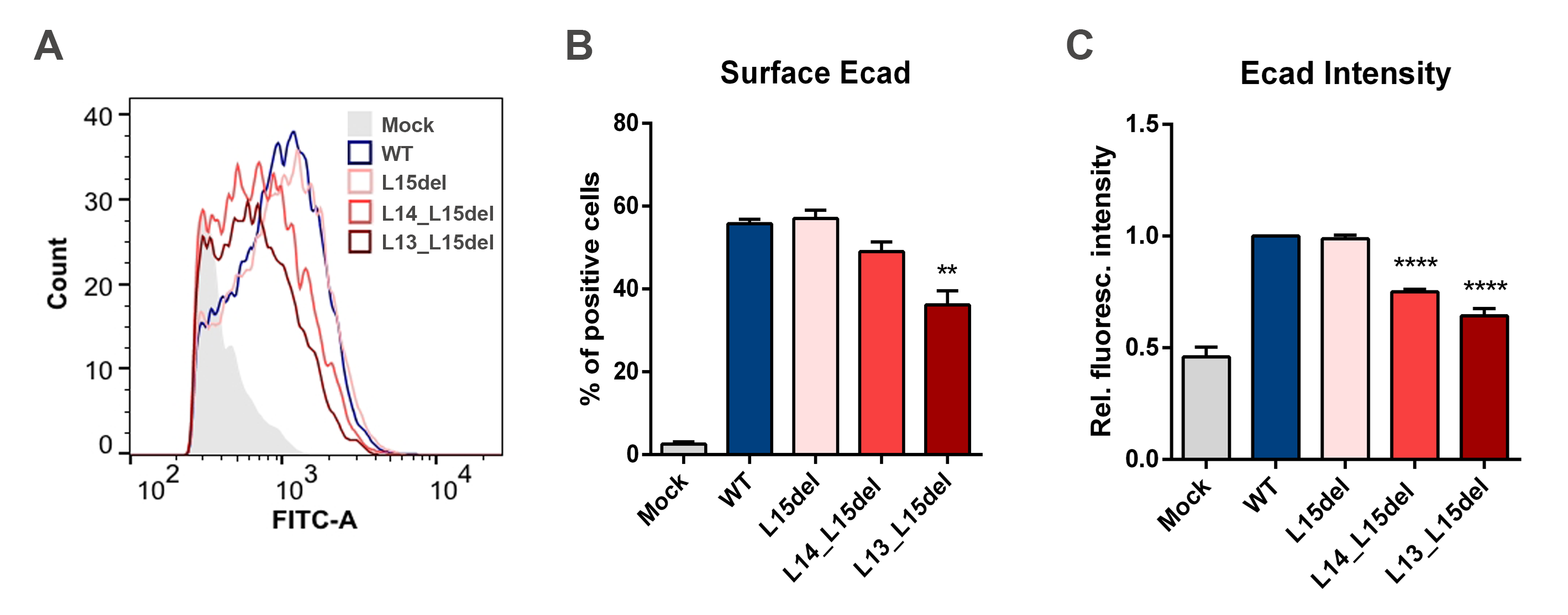

Supplement: Supplementary file 4 — Figure S2. E-cadherin surface expression induced by the p.L15del, p.L14_L15del and p.L13_L15del variants. (A) Histogram showing E-cadherin surface expression in cells transfected with plasmids encoding the wild-type or the L15del, L14_L15del and L13_L15del E-cadherin mutant forms. Mock cells were used as a negative control. (B) Percentage of cells expressing E-cadherin at the plasma membrane. The graph shows the average + SE of four independent experiments. (C) The relative median fluorescence intensity was determined in each cell line. * represents p ≤ 0.05, ** p ≤ 0.01, *** p ≤ 0.001 and **** p ≤ 0.0001. (TIF 496 kb) [file 12943_2018_859_MOESM4_ESM.tif]
