# Supplementary material for: E-cadherin signal sequence disruption: a novel mechanism underlying hereditary cancer
Source: Mol Cancer. 2018 Aug 1;17:112. doi: 10.1186/s12943-018-0859-0 (PMC6090902; doi:10.1186/s12943-018-0859-0)
Supplement: Supplementary file 3 — Methods and Materials. (DOCX 44 kb) [file 12943_2018_859_MOESM3_ESM.docx]

**Methods**

*DNA extraction and variant detection* – Genomic DNA was extracted from peripheral blood using the QIAamp DNA blood Mini Kit (Qiagen), according to the manufacturer’s instructions. Mutation analysis was performed by Sanger sequencing of coding and flanking intronic sequence of exon 1. Bi-directional sequencing was carried out using BigDye Terminator v3.1 Cycle Sequencing Kit (Thermo Fisher Scientific) and analyzed on an ABI 3730 DNA analyzer (Applied Biosystems). Resulting sequences were compared with the reference DNA (ENSG00000039068) by the Mutation Surveyor software (SoftGenetics LLC, version 4.05). Variant nomenclature is in accordance with the Human Genome Variation Society (HGVS) guidelines.

*Plasmids* – The E-cadherin variant L13_L15del (c.38_46del) was generated in the pIRES2-EGFP vector (Clontech, Takara Bio) encoding the Human E-cadherin cDNA (2649 bp, ENSP00000261769). For the *in vitro* translation experiments, the L13_L15del (c.38_46del), L14_L15del (c.41_46del) and L15del (c.44_46del) variants were induced downstream the T7 RNA polymerase promoter in the CDH1pEF6/Myc-His vector previously established by our group [[1](#_ENREF_1)]. The corresponding empty vectors (Mock) were used as a control. All the clonings were verified by direct sequencing.

*Cell culture and transfection –* CHO cell line (Chinese Hamster Ovary, ATCC number: CCL-61) was cultured in α-MEM medium (Gibco, Invitrogen) supplemented with 10% fetal bovine serum (HyClone, Perbio) and 1% penicillin/streptomycin (Gibco, Invitrogen). Cells were maintained at 37°C under 5% CO_2_ humidified air. For transfection, 1.6 X 10^5^ cells were seeded in 6-well plates and, 24h later, cells were transiently transfected with vectors encoding either the wild-type protein or the variant L13_L15del, as well as the empty vector (Mock condition). Transfections were carried out using Lipofectamine 2000 (Invitrogen), following the manufacturer's recommendations. Transfection efficiency was evaluated in each experiment by the assessment of GFP positive cells through flow cytometry.

*Cell treatments* – Thirty six hours after transfection, cells were treated with 2% dimethyl sulfoxide (DMSO; Sigma) for 24h. The proteasome inhibitor MG132 (Calbiochem) was used at 10µM concentration for 17h.

*Fluorescence-activated cell sorting (FACS)* *–* For flow cytometry analysis, cells were detached with Versene (Gibco, Invitrogen) and resuspended in ice cold PBS with 0.05 mg/ml CaCl_2_. 5 X 10^5^ cells from the suspension were incubated for 1h with the extracellular primary antibody HECD1 (Invitrogen) at 1:50 dilution. Cells were washed twice and, subsequently, incubated with Alexa Fluor 594 goat anti-mouse (1:250, Invitrogen) in the dark for 30 minutes. Finally, the cells were washed and resuspended in 0.5ml of washing solution. Acquisition was performed in FACS Aria II and data was analyzed with FlowJo software version 10.08.

*Western blotting –* Lysates were prepared by scraping the cells in cold Catenin Buffer [1% Triton X-100 (Sigma) and 1% IGEPAL CA-630 (Sigma) in PBS], supplemented with a cocktail of phosphatase (Sigma) and protease inhibitors (Roche). Protein concentration was assessed using a modified Bradford assay (Bio-Rad). For analysis, 15μg of total protein were eluted in sample buffer, separated in 7.5% SDS-polyacrylamide gel electrophoresis (SDS–PAGE) and electroblotted onto Hybond ECL membranes (Amersham Biosciences). Membranes were blocked in 5% non-fat milk and 0.5% Tween-20 in PBS for 1h, and thereafter immunoblotted with antibodies against E-cadherin (1:2500, Clone HECD1 Invitrogen) and α-Tubulin (1:10000, Sigma). The secondary antibody sheep anti-mouse HRP-conjugated (Amersham Biosciences) was applied, followed by detection with ECL reagents (Bio-Rad). Protein bands were quantified by densitometry using the Quantity One Software (Bio-Rad).

*E-cadherin immunofluorescence –* Cells seeded on top of glass coverslips were washed in PBS and fixed in ice-cold methanol for 20 minutes. Blocking was performed with 3% BSA in PBS for 30 minutes, at room temperature. Cells were incubated with an E-cadherin mouse monoclonal antibody (BD Biosciences) diluted at 1:300 in the blocking solution, for 1h. The secondary antibody Alexa Fluor 488 goat anti-mouse (1:500, Invitrogen) was then applied for 1h in the dark. Coverslips were mounted on slides using Vectashield with DAPI (Vector Laboratories). Image acquisition was performed on a Carl Zeiss Apotome Axiovert 200M Fluorescence Microscope with an Axiocam HRm camera, and processing done with the Zeiss Axion Vision 4.8 software.

*Expression profiling –* The networks obtained for topological analysis were also used for extraction of a quantitative picture of E-cadherin distribution along two contiguous cells (internuclear profiles). The intensity of fluorescent signals that occur in segments connecting two neighbouring nuclei was calculated/measured as described by Sanches et al. [[2](#_ENREF_2)]. Position 1 corresponds to the geometric center of nucleus 1, position 100 is the center of nucleus 2, and position 50 represents the plasma membrane. Signal intensity of each position from 1 to 100 was obtained and statistically examined.

*Matrigel invasion assay* – Matrigel invasion chambers suitable for 24-well-plates (Corning BioCoat) were used to evaluate cell invasive properties. Inner and outer compartments of matrigel inserts were first hydrated with α-MEM medium for 1h at 37ºC. Thereafter, 500µl of a cellular suspension of 5 x 10^4^cells/ml (containing 2.5 x 10^4^cells) were plated in each chamber, and the plate was incubated at 37ºC in a humidified atmosphere with 5% CO_2_. 24h after seeding, non-invasive cells were removed from the upper chamber with a pre-wet ‘cotton swab’. The filters were washed in PBS, fixed in ice-cold methanol for 15 minutes, and mounted in slides with Vectashield medium with DAPI (Vector Laboratories). The total number of invasive nuclei present in the bottom of each filter was counted under a Leica DM2000 microscope.

*Slow aggregation assay* – Wells of 96-well-plates were coated with 50µl of an agar solution, prepared by solving 100mg of Bacto-Agar in 15ml of sterile PBS [[1](#_ENREF_1), [3](#_ENREF_3)]. Once the agar solidifies, 200µl of a cellular suspension of 1 x 10^5^cells/ml (corresponding to 2 x 10^4^cells) were added to the coated wells. Each condition was evaluated in triplicate. The plate was incubated at 37°C and 5% CO_2_ humidified air for 48h. Cell-cell adhesion phenotypes were evaluated and photographed under a Leica DMi1 inverted microscope with camera, 24h and 48h after seeding. For quantification purposes, the area of cellular aggregates present in the images was assessed in ImageJ 1.49q.

*Real-time PCR* – Cell total RNA was isolated using TripleXtractor (Grisp), according to the manufacturer's instructions. cDNA was synthesized from 1µg of RNA with qScript XLT cDNA SuperMix (Quantabio). *CDH1* expression was evaluated using the TaqMan probe Hs01023895_m1 (Thermo Fisher Scientific) and normalized to the levels of 18S (Hs99999901_s1, Thermo Fisher Scientific). Data was analyzed using ΔΔCt method in an ABI Prism 7500 Fast System (Applied Biosystems).

*In vitro transcription and translation* – Combined transcription and translation of wild-type and mutant cDNAs was carried out using the TnT Quick Coupled Transcription/Translation System (Promega) specific for genes cloned downstream from the T7 RNA polymerase promoter. For the reactions, 1µg of each circular plasmid DNA was added to the TnT Quick Master Mix and incubated for 90 minutes at 30°C. The synthesized proteins were then detected by SDS-PAGE analysis as described above (Western blotting protocol).

*Statistical analysis –* Statistical data analysis was performed using the two-tailed unpaired Student's *t*-test from GraphPad Prism software, and *p*≤0.05 was required for significance. The Wilcoxon signed-rank test was applied for internuclear profile analysis.

*Multiple sequence alignment –* Peptides sequences from human E-cadherin (ENSP00000261769), chimpanzee E-cadherin (ENSPTRP00000014119), mouse E-cadherin (ENSMUSP00000000312), *Xenopus* E-cadherin (ENSXETP00000035755) and human P-cadherin (ENSP00000398485) were collected from Ensembl browser (https://www.ensembl.org/index.html). Sequence variation was analysed through ClustalW 2.1 program from Galaxy platform using default settings. Image was prepared with GeneDoc version 2.2.0.

*In silico predictions* – PROVEAN (Protein Variation Effect Analyzer, <http://provean.jcvi.org/index.php>) was applied to predict the impact of the variant on protein function [[4](#_ENREF_4)]. The algorithm was run with the Ensembl transcript 261769, mutation p.L13_L15del, p.L14_L15del and p.L15del. SignalP 4.1 (http://www.cbs.dtu.dk/services/SignalP/) was used to evaluate the impact of the mutation in the location of signal peptide cleavage sites [[5](#_ENREF_5)]. This software integrates the prediction of cleavage sites and the prediction of signal/non-signal peptides based on a combination of several artificial neural networks [[5](#_ENREF_5)]. A previously described scoring function was then applied to SignalP data to infer functional consequences in the signal peptide of E-cadherin variants [[6](#_ENREF_6)].

**References**

1. Figueiredo J, Soderberg O, Simoes-Correia J, Grannas K, Suriano G, Seruca R: **The importance of E-cadherin binding partners to evaluate the pathogenicity of E-cadherin missense mutations associated to HDGC.** *Eur J Hum Genet* 2013, **21:**301-309.

2. Sanches JM, Figueiredo J, Fonseca M, Duraes C, Melo S, Esmenio S, Seruca R: **Quantification of mutant E-cadherin using bioimaging analysis of in situ fluorescence microscopy. A new approach to CDH1 missense variants.** *Eur J Hum Genet* 2015, **23:**1072-1079.

3. Suriano G, Oliveira C, Ferreira P, Machado JC, Bordin MC, De Wever O, Bruyneel EA, Moguilevsky N, Grehan N, Porter TR, et al: **Identification of CDH1 germline missense mutations associated with functional inactivation of the E-cadherin protein in young gastric cancer probands.** *Hum Mol Genet* 2003, **12:**575-582.

4. Choi Y, Chan AP: **PROVEAN web server: a tool to predict the functional effect of amino acid substitutions and indels.** *Bioinformatics* 2015, **31:**2745-2747.

5. Petersen TN, Brunak S, von Heijne G, Nielsen H: **SignalP 4.0: discriminating signal peptides from transmembrane regions.** *Nat Methods* 2011, **8:**785-786.

6. Hon LS, Zhang Y, Kaminker JS, Zhang Z: **Computational prediction of the functional effects of amino acid substitutions in signal peptides using a model-based approach.** *Hum Mutat* 2009, **30:**99-106.
